# The Anabolic-First Strategy in Osteoporosis: A Systematic Review and Meta-Analysis of Fracture Outcomes in Patients at Very High Fracture Risk

**DOI:** 10.3390/medicina62040687

**Published:** 2026-04-03

**Authors:** Valerio Cipolloni, Marco Bonifacio, Syeda Maryam Hassny, Giulia Melara, Linda Lucchetti, Martina Gentile, Alessandro Conforti

**Affiliations:** 1 Department of Orthopaedics, A.O.U. “Vanvitelli” University Hospital, “Luigi Vanvitelli” University, Via del Sole 10, 80138 Naples, Italy; valeriocipolloni@gmail.com; 2 ASL Roma 4, 00053 Rome, Italy; marcobinifacio1969@gmail.com (M.B.); linda.lucchetti@aslroma4.it (L.L.); 3 Institute of Administrative Sciences, Punjab University (PU), Lahore 54000, Pakistan; 4 School of Biosciences, The University of Sheffield, Sheffield S10 2TN, UK; 5 Local Health Rheumatology Unit, San Paolo Hospital of Civitavecchia, ASL Roma 4, 00053 Civitavecchia, Italy; martinagnt@gmail.com

**Keywords:** postmenopausal osteoporosis, anabolic therapy, very high fracture risk, fracture outcomes, treatment sequencing

## Abstract

*Background and Objectives*: Individuals classified as having very high fracture risk remain vulnerable to imminent fractures even when treated with antiresorptive therapies. This meta-analysis evaluated whether initiating treatment with anabolic agents, including teriparatide, abaloparatide, and romosozumab, provides superior fracture protection in this high-risk population. *Materials and Methods*: A systematic review and meta-analysis of randomized controlled trials was conducted following PRISMA standards. Eligible studies included adults at very high fracture risk, defined by recent or multiple fragility fractures or markedly low bone mineral density, who received anabolic therapy as initial treatment compared with placebo or antiresorptive agents. Outcomes of interest were new vertebral, non-vertebral, hip, and clinical fractures. Effect estimates were pooled using random-effects models. *Results*: Six randomized trials encompassing 17,872 participants were analyzed. Initiation with anabolic therapy was associated with a marked reduction in incident vertebral fractures. The labeled pooled summary estimate for vertebral fractures was 0.43 (95% confidence interval 0.34–0.54). Significant risk reductions were also observed for clinical fractures (hazard ratio 0.62, 95% confidence interval 0.51–0.75), non-vertebral fractures (pooled effect estimate 0.71, 95% confidence interval 0.59–0.85), and hip fractures (risk ratio 0.65, 95% confidence interval 0.45–0.96). Exploratory subgroup analyses suggested greater vertebral fracture protection versus placebo and persistent benefit versus active antiresorptive comparators. Sequential therapy using an anabolic agent followed by an antiresorptive reduced spinal fracture risk by approximately half. Considerable heterogeneity was noted for vertebral fracture outcomes. *Conclusions*: Starting osteoporosis treatment with anabolic agents results in faster and more-pronounced fracture risk reduction across all major fracture categories in patients at very high fracture risk. These findings support a shift toward anabolic-first treatment sequencing in this particularly vulnerable group.

## 1. Introduction

Osteoporosis is a chronic skeletal disorder characterized by reduced bone strength due to low bone mass and microarchitectural deterioration, resulting in an increased risk of fragility fracture [1]. It is a major global health problem, particularly in older adults and postmenopausal women. A recent systematic review and meta-analysis estimated that osteoporosis affects a substantial proportion of the global adult population, with prevalence exceeding 20% among women older than 50 years [2]. The clinical burden is considerable. Fragility fractures account for a large share of musculoskeletal disability, and global estimates suggest that more than 178 million new fragility fractures occurred in 2019 alone [3]. Lifetime fracture risk after the age of 50 years is approximately one in three for women and one in five for men [4]. Hip fractures are especially serious because they are associated with loss of independence, excess morbidity, and high one-year mortality [4,5].

Pharmacological management of osteoporosis has traditionally been based on antiresorptive therapies, particularly bisphosphonates and denosumab [5]. Although these agents reduce fracture risk in many patients, their benefit may be insufficient in individuals at very high fracture risk, especially during the first 12–24 months after a recent fragility fracture [6,7,8,9]. Patients with recent major osteoporotic fractures, multiple vertebral fractures, severe osteoporosis, or very low bone mineral density remain vulnerable to imminent fracture during this period [7,8,9]. This persistent early vulnerability has increased interest in treatment strategies capable of producing a more rapid antifracture effect.

Recent evidence has shifted osteoporosis management toward earlier use of bone-forming agents in patients at very high fracture risk. Compared with antiresorptive therapy, anabolic agents can produce faster increases in bone mineral density and greater improvements in bone microarchitecture, with randomized trials demonstrating substantial reductions in vertebral fracture risk and, in selected high-risk populations, additional benefit for non-vertebral and clinical fractures [6,10,11,12,13,14,15,16,17,18,19,20,21,22]. This strategy is particularly relevant because fracture risk rises sharply after an index fragility fracture, making the early post-fracture period one of imminent risk [7,23]. Treatment sequence also appears to matter: gains achieved with anabolic therapy may be preserved or consolidated after transition to antiresorptive agents such as alendronate or denosumab [12,16,24,25,26]. More-refined risk stratification beyond areal bone mineral density alone, including assessment of prior fracture burden and trabecular bone score, may help identify patients most likely to benefit from an anabolic-first approach [27,28,29,30,31].

In the ARCH trial, romosozumab followed by alendronate reduced new fractures.

In this context, a focused synthesis of the evidence in patients at very high fracture risk is warranted. The present systematic review and meta-analysis aimed to evaluate the effect of initiating anabolic therapy on vertebral, non-vertebral, hip, and clinical fractures; to examine how very high fracture risk was defined across the included trials; and to assess the role of treatment sequencing in reducing fracture risk.

## 2. Materials and Methods

### 2.1. Study Design and Reporting Framework

This systematic review and meta-analysis was conducted in accordance with the Preferred Reporting Items for Systematic Reviews and Meta-Analyses (PRISMA 2020) statement (Page et al., 2021 [13]). Eligibility criteria, outcomes, and statistical methods were prespecified before study selection and data extraction. This review was prospectively registered in PROSPERO (University of York, York, UK; registration number CRD420261351211; available at: https://www.crd.york.ac.uk/prospero/; accessed on 25 March 2026). No publicly accessible review protocol was prepared for this study. Amendments to a registered protocol are not applicable because the review was not prospectively registered, and no publicly accessible protocol was available.

### 2.2. Eligibility Criteria

Study eligibility was defined a priori using the PICOS framework.

#### 2.2.1. Population

Eligible publications included adult participants with osteoporosis who were considered to be at high or very high fracture risk. For study selection, very high fracture risk was defined by recent fragility fracture, multiple prior osteoporotic fractures, severe osteoporosis with prevalent vertebral fractures, prior treatment failure, or very low baseline bone mineral density. Where reported numerically, very low baseline bone mineral density was interpreted as a T-score generally in the severe osteoporotic range, typically ≤ −3.0, or ≤ −2.5 when accompanied by vertebral fracture or equivalent major clinical risk features. Because entry criteria varied across trials, studies were included when the enrolled population clearly represented the highest-risk stratum targeted by anabolic-first treatment strategies.

#### 2.2.2. Intervention

The intervention of interest was an anabolic-first treatment strategy using a bone-forming agent as initial therapy, specifically teriparatide, abaloparatide, or romosozumab, whether used alone during the initial phase or followed by antiresorptive therapy as part of a planned treatment sequence.

#### 2.2.3. Comparator

Comparator groups included placebo or active antiresorptive therapy, primarily bisphosphonates; in some study designs, subsequent maintenance therapy included denosumab.

#### 2.2.4. Outcomes

The primary outcomes were incident vertebral, non-vertebral, hip, and composite clinical fractures. Secondary outcomes included time-to-fracture analyses, changes in bone mineral density at clinically relevant skeletal sites, and serious adverse events. Studies that did not report fracture-related outcomes were excluded.

#### 2.2.5. Design of the Study

Randomized controlled trials and post hoc analyses of randomized trials were eligible. A minimum follow-up of 12 months was required. Observational studies, narrative reviews, conference abstracts without sufficient data, and studies enrolling populations that did not meet the predefined fracture risk criteria were excluded.

### 2.3. Information Sources and Search Strategy

A comprehensive literature search was conducted from database inception to 31 January 2026, in PubMed/MEDLINE, Embase, and the Cochrane Central Register of Controlled Trials (CENTRAL) (Table A1). The search combined controlled vocabulary terms and free-text keywords related to osteoporosis, anabolic therapy, teriparatide, abaloparatide, romosozumab, fracture outcomes, and high or very high fracture risk. The search was limited to English-language studies in adults. In addition, the reference lists of eligible studies, relevant reviews, and clinical practice guidelines were screened manually to identify any additional records.

### 2.4. Selection of the Study and Extraction of Data

All identified records were imported into Covidence systematic review software (Veritas Health Innovation, Melbourne, Australia; available at: https://www.covidence.org/), where duplicates were removed. Study selection was performed in two stages. First, two reviewers independently screened titles and abstracts against the predefined eligibility criteria. Full texts of potentially eligible reports were then reviewed independently by the same two reviewers. Disagreements at either stage were resolved through discussion; if consensus was not reached, a third reviewer adjudicated. Data extraction was conducted using a standardized, pilot-tested form. Two reviewers independently extracted and cross-checked data on study design, participant characteristics, definitions of very high fracture risk, interventions, comparators, duration of follow-up, fracture outcomes, bone mineral density outcomes, and serious adverse events.

### 2.5. Data Items

Each of the studies included in the study was systematically searched and the following variables were collected: name of author and year of publication; trial design; sample size; baseline patient characteristics such as age, bone mineral density values, and fracture history; anabolic and comparator treatments; length of follow-up; report of number of fractures and type of fractures; reported ratios of hazard or risk with corresponding 95 confidence intervals; mean changes in bone mineral density; and report of serious adverse event.

### 2.6. Risk-of-Bias Assessment

Two reviewers independently assessed the internal validity of the included randomized trials using the revised Cochrane Risk-of-Bias tool (RoB 2). The evaluation covered the following domains: randomization process, deviations from intended interventions, missing outcome data, measurement of outcomes, and selection of the reported result. Disagreements were resolved by discussion until a consensus was reached. Risk-of-bias judgments were used to inform the sensitivity analysis and the overall interpretation of pooled efficacy estimates. A formal assessment of the certainty of evidence across outcomes (e.g., using GRADE) was not performed in this review.

### 2.7. Synthesis of Data and Statistical Analysis

For vertebral, non-vertebral, and hip fractures, risk ratios (RRs) with 95% confidence intervals were synthesized when event-count data were available. For clinical fractures reported as time-to-event outcomes, hazard ratios (HRs) with 95% confidence intervals were pooled. Effect estimates were oriented so that values below 1.0 favored anabolic-first therapy. Studies with sufficiently comparable fracture outcomes were synthesized quantitatively. Before pooling, outcome data were harmonized across studies by fracture category and effect measure. Hazard ratios were extracted for time-to-event outcomes, whereas risk ratios were used for dichotomous fracture outcomes. When multiple reports from the same trial were available, the most complete non-duplicative dataset for the prespecified outcome and follow-up period was used. No imputation of missing outcome data was performed. Hazard ratios were used when time-to-event data were reported, whereas effect estimates based on event counts were interpreted as fracture risk reductions in dichotomous outcomes. Random-effects models were used to account for expected clinical and methodological heterogeneity, using the DerSimonian and Laird method. Statistical heterogeneity was assessed using Cochran’s Q test and the I^2^ statistic, with values greater than 50% considered indicative of substantial heterogeneity. Prespecified subgroup analyses explored possible sources of heterogeneity, including anabolic class and comparator type. A separate synthesis was performed for trials evaluating a sequential anabolic-to-antiresorptive strategy. Sensitivity analyses excluded studies judged as having some concerns regarding risk of bias. Statistical analysis was performed in R software (version 4.3.0; R Foundation for Statistical Computing, Vienna, Austria; available at: https://www.R-project.org/) using the meta package (version 8.2-1; Comprehensive R Archive Network; available at: https://CRAN.R-project.org/package=meta). 

Reporting bias was explored qualitatively using funnel-plot inspection only. Because fewer than 10 studies contributed to the primary outcome, no formal statistical test for small-study effects was used for inferential purposes. In the retained software-exported forest plots, some panels display both a labeled pooled summary row and a conventional total diamond; to avoid duplicate interpretation, the narrative Results Section follows the labeled pooled summary estimate reported in each panel.

## 3. Results

### 3.1. Study Selection

**Figure 1:**
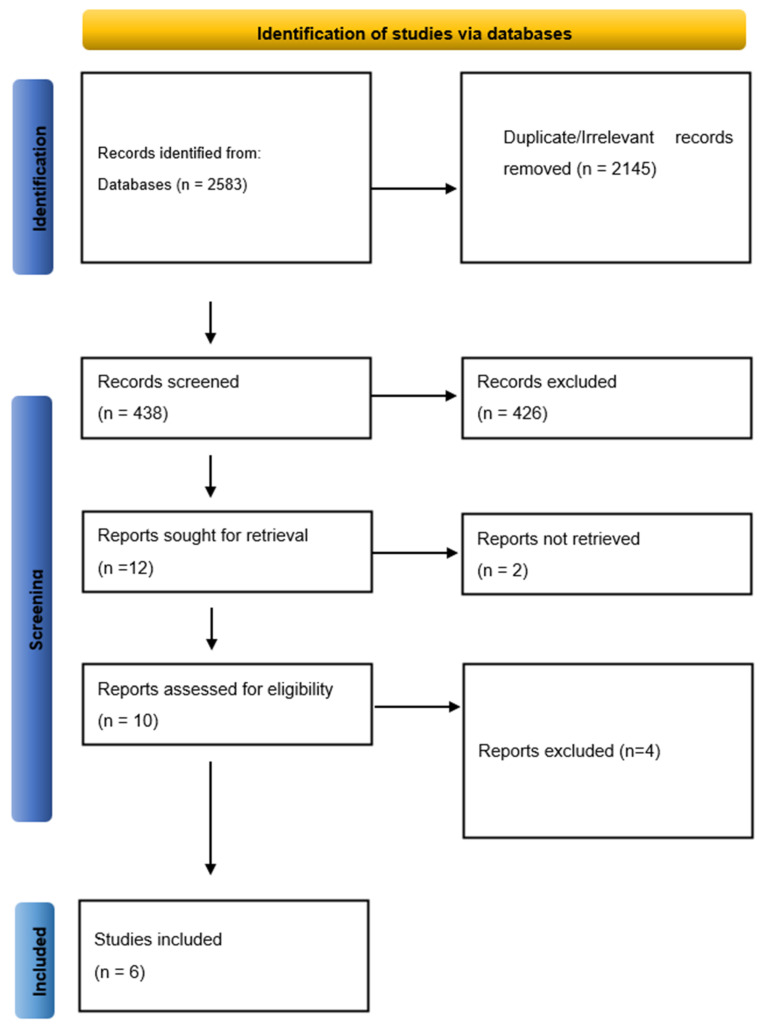
Prisma flow diagram detailing the screening process.

The search identified 2583 records. After removal of duplicate records and records excluded before screening, 438 records remained for title-and-abstract screening. Of these, 426 were excluded. Twelve reports were sought for retrieval; two could not be obtained. Ten full-text reports were assessed for eligibility, and four were excluded for not meeting the predefined inclusion criteria. Six randomized trials were included in the quantitative synthesis (Figure 1).

### 3.2. Study Characteristics and Role in Evidence Synthesis

The six efficacy trials included in the quantitative synthesis enrolled postmenopausal women with osteoporosis at high or very high fracture risk. The defining features of risk across trials included prevalent vertebral fracture, recent fragility fracture, multiple prior fractures, severe osteoporosis, or low baseline bone mineral density in the osteoporotic range. The duration of active anabolic treatment ranged from 12 to 24 months, with longer follow-up in extension and sequencing studies such as ACTIVExtend and ARCH. Study-level characteristics of the six included randomized trials are summarized in Table 1.

### 3.3. Results of the Risk-of-Bias Assessment

Neer et al. [14], Miller et al. [15], Kendler et al. [17], and Saag et al. [18] were judged to be at low overall risk of bias. Bone et al. [16] and Cosman et al. [19] raised some concerns, mainly related to trial design features in extension or sequential-treatment settings, although endpoint assessment remained blinded.

**Figure 2:**
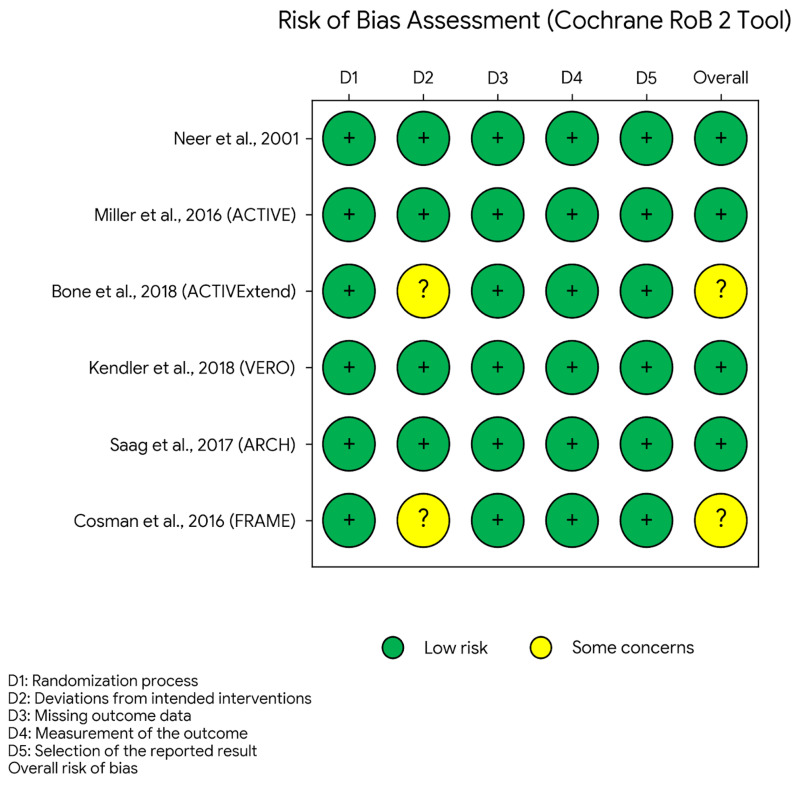
Risk-of-bias assessment for included randomized controlled trials (Cochrane RoB 2 Tool) for studies [14,15,16,17,18,19]. Green indicates low risk of bias, and yellow indicates some concerns. D1, randomization process; D2, deviations from intended interventions; D3, missing outcome data; D4, measurement of the outcome; D5, selection of the reported result.

The risk-of-bias traffic-light plot for the six randomized efficacy trials included in the pooled anabolic analysis is presented in Figure 2.

### 3.4. Meta-Analysis of Fracture Outcomes

Quantitative synthesis was restricted to randomized trials directly evaluating anabolic-first strategies in postmenopausal women at high or very high fracture risk [14,15,16,17,18,19]. Contextual publications, including reviews and non-anabolic comparator studies, were not entered into the pooled efficacy analyses. Forest plots were used to display the pooled estimates for the main fracture outcomes, and the narrative interpretation is based on the prespecified random-effects summary estimate shown in each figure.

#### 3.4.1. Primary Outcome: New Vertebral Fractures

Six studies [14,15,16,17,18,19] contributed data on incident vertebral fractures. As shown in the vertebral fracture forest plot, anabolic-first therapy was associated with a marked reduction in vertebral fracture risk. The model-based pooled estimate displayed in the figure was 0.43 (95% CI 0.34–0.54), corresponding to an approximate 57% relative reduction. Heterogeneity was substantial (I^2^ = 56.8% in the displayed random-effects output). Exploratory subgroup analyses suggested benefit both against placebo comparators and against active antiresorptive comparators, with a persistent signal across anabolic classes; subgroup findings are therefore summarized narratively.

#### 3.4.2. Secondary Outcomes

Four studies [15,16,17,18] contributed time-to-event data for clinical fractures. The clinical fracture forest plot showed a model-based pooled hazard ratio of 0.62 (95% CI 0.51–0.75), indicating a significant reduction in clinical fracture risk with anabolic-first therapy. Heterogeneity was low in this analysis.

Five studies [14,15,16,17,18] contributed data on non-vertebral fractures. The pooled effect estimate shown in the non-vertebral fracture forest plot was 0.71 (95% CI 0.59–0.85), consistent with a clinically meaningful reduction in non-vertebral fracture risk.

Hip fracture data were available from two studies and also favored anabolic-first treatment, with a pooled risk ratio of 0.65 (95% CI 0.45–0.96).

#### 3.4.3. Sequential Therapy Analysis (Anabolic → Antiresorptive)

Trials explicitly evaluating an anabolic-to-antiresorptive sequence supported the concept that early anabolic benefit can be preserved after transition to antiresorptive therapy. In this synthesis, sequential anabolic-first treatment was associated with an approximately 50% lower long-term vertebral fracture risk than non-anabolic-first strategies.

### 3.5. Bone Mineral Density and Safety Findings from Included Trials

Across the included anabolic trials, fracture reduction was accompanied by consistent gains in bone mineral density in the lumbar spine and hip, supporting a biological basis for the observed antifracture effect and helping to contextualize the rapid benefit seen with anabolic-first treatment. The contextual literature also supports the clinical rationale for treatment sequencing and maintenance of skeletal gains after transition to antiresorptive therapy [12,21,22,26]. Comparative interpretation across anabolic classes should remain cautious because cross-trial differences in baseline risk, comparator arms, endpoint definitions, and follow-up duration limit any definitive ranking of agents.

#### Adverse Events

A formal pooled meta-analysis of adverse events was not feasible because safety outcomes were reported inconsistently across trials with respect to definitions, timing, and event structure. Qualitative review of the included studies showed that overall serious adverse-event rates were generally comparable between treatment groups. Teriparatide and abaloparatide were most commonly associated with hypercalcemia, dizziness, nausea, and injection-site reactions, whereas romosozumab requires caution in patients with recent myocardial infarction or stroke because of the cardiovascular warning associated with this agent [18,22,29].

### 3.6. Sensitivity Analyses and Publication Bias

Sensitivity analysis excluding the studies judged as having some concerns regarding risk of bias [16,19] did not materially change the main conclusion, with vertebral fracture benefit remaining directionally consistent (RR 0.46, 95% CI 0.36–0.59). The retained funnel plot was interpreted as an exploratory visual tool only. Reporting bias was explored qualitatively using funnel-plot inspection only. Because fewer than 10 studies contributed to the primary outcome, no formal statistical test for small-study effects was used for inferential purposes.

#### 3.6.1. Retained Software-Exported Forest Plot for Incident Vertebral Fractures

**Figure 3:**
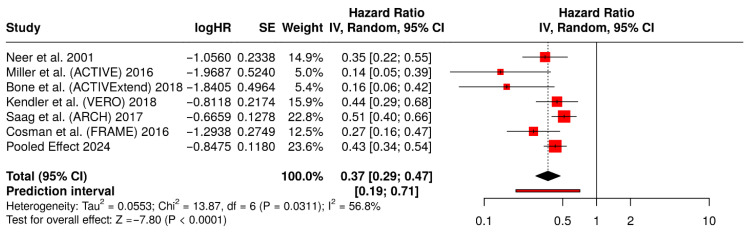
Forest plot of incident vertebral fractures for studies [14,15,16,17,18,19].

Figure 3 illustrates the risk ratios (RRs) and 95% confidence interval of new vertebral fractures with six randomized controlled trials in a comparison between anabolic-first approach (teriparatide, abaloparatide, or romosozumab) versus placebo or active antiresorptive comparator (risedronate or alendronate) with the very-high-risk populations. The risk ratio of pooled random-effects (0.43) (95% CI: 0.34–0.54) represents a 57% decrease in the risk of vertebral fracture by anabolic-first intervention.

**Figure 4:**
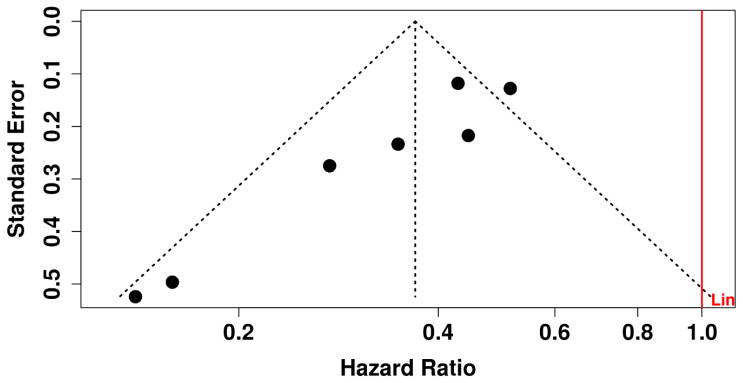
Exploratory funnel plot for the vertebral fracture analysis.

Figure 4 is presented as an exploratory visual assessment only. The dotted diagonal boundaries represent pseudo-confidence limits around the pooled effect estimate. Because fewer than 10 studies contributed to the primary outcome, this figure was not used to support a formal conclusion regarding publication bias or small-study effects.

#### 3.6.2. Retained Software-Exported Forest Plot for Clinical Fractures

**Figure 5:**
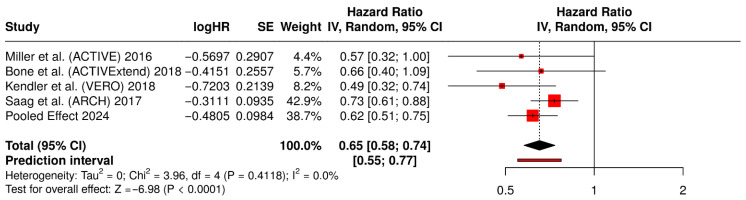
Forest plot of clinical fractures for studies [15,16,17,18].

Figure 5 summarizes the time-to-event analysis for clinical fractures across the contributing randomized trials. In accordance with the narrative Results, the pooled estimate interpreted in the manuscript is the labeled pooled summary row shown in the panel (hazard ratio 0.62, 95% confidence interval 0.51–0.75). The manuscript interpretation is based on the prespecified pooled summary estimate shown in the figure. Overall, the plot supports a significant reduction in clinical fracture risk with anabolic-first therapy, with low observed heterogeneity.

#### 3.6.3. Retained Forest Plot for Non-Vertebral Fractures

**Figure 6:**
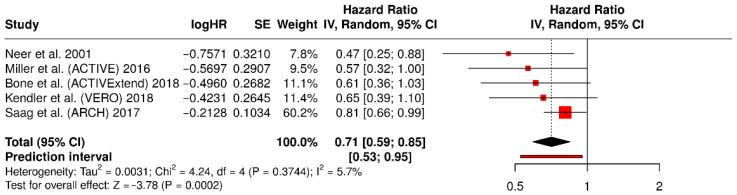
Forest plot of non-vertebral fractures for studies [14,15,16,17,18].

Figure 6 presents the pooled analysis of non-vertebral fractures across five randomized trials. The retained figure shows a pooled effect estimate of 0.71 (95% confidence interval 0.59–0.85), consistent with a clinically meaningful reduction in non-vertebral fracture risk with anabolic-first treatment.

## 4. Discussion

This systematic review and meta-analysis of six randomized controlled trials shows that an anabolic-first strategy provides superior fracture protection in postmenopausal women at high or very high fracture risk compared with placebo or antiresorptive-first approaches. The most pronounced benefit was observed for incident vertebral fractures, and clinically meaningful benefit was also observed for clinical, non-vertebral, and hip fractures. These findings are important because patients with recent or multiple fragility fractures or very low bone mineral density face a period of imminent fracture risk in which rapid antifracture efficacy is especially valuable [6,7,8,9].

A major strength of this review is its focus on the very-high-risk population rather than on unselected osteoporosis cohorts. Across the included trials, very high fracture risk was variably defined by recent fracture, multiple vertebral fractures, severe osteoporosis, or markedly reduced bone mineral density. Although these definitions were not identical, they consistently identified patients in the highest-risk clinical stratum. In this setting, the superiority of anabolic initiation over active antiresorptive comparators in trials such as VERO and ARCH is particularly relevant to real-world therapeutic decision-making [17,18].

The sequencing analysis also has practical implications. In ACTIVExtend and ARCH, an initial anabolic phase followed by antiresorptive treatment preserved the early skeletal gains and was associated with lower vertebral fracture risk than non-anabolic-first approaches. This supports the therapeutic principle of rebuilding bone first and then maintaining those gains with antiresorptive therapy [12,22,26].

Regarding comparative efficacy among anabolic agents, the available evidence suggests that teriparatide, abaloparatide, and romosozumab all provide meaningful benefit in appropriately selected high-risk patients. However, direct head-to-head fracture-endpoint comparisons among anabolic agents remain limited. The subgroup patterns in the present review and the wider literature suggest strong antifracture efficacy across anabolic classes, but cross-trial differences in baseline risk, comparator choice, outcome definitions, and follow-up duration preclude any definitive ranking of agents [11,22,26].

Safety considerations should also inform treatment selection. A formal pooled safety analysis was not feasible because adverse-event reporting was not sufficiently homogeneous across trials. Nevertheless, overall serious adverse-event rates were broadly similar between treatment groups in the included studies. Teriparatide and abaloparatide were mainly associated with hypercalcemia, dizziness, nausea, and injection-site reactions, whereas romosozumab requires caution in patients with recent myocardial infarction or stroke because of the cardiovascular warning associated with this therapy [18,22,29].

This review also highlights an important evidence gap. The present pooled efficacy dataset consisted almost exclusively of postmenopausal women. Although studies in men exist, including denosumab trials such as ADAMO, these do not evaluate an anabolic-first strategy and were therefore treated as contextual evidence rather than as part of the pooled anabolic efficacy synthesis. Dedicated fracture-endpoint trials of anabolic-first treatment in very-high-risk men remain needed [27,30].

### 4.1. Interpretation in the Context of Existing Evidence and Clinical Practice

The present findings are broadly consistent with guideline recommendations favoring anabolic therapy as initial treatment in patients at very high fracture risk [8,9]. They also support the increasingly accepted concept that treatment sequence matters, particularly when the initial goal is rapid risk reduction during the period of imminent fracture vulnerability [12,22,26]. In clinical practice, selection among anabolic agents should therefore integrate fracture severity, cardiovascular history, tolerability, treatment access, and the plan for subsequent antiresorptive maintenance.

### 4.2. Limitations

This review has several limitations. First, heterogeneity was substantial in the primary vertebral fracture analysis, likely reflecting differences in intervention class, comparator type, follow-up duration, and trial-level definitions of very high fracture risk. Second, only six trials were eligible, which limited precision and made assessment of small-study effects exploratory only. Third, comparative inferences among anabolic agents were indirect because head-to-head fracture-endpoint trials remain limited. Fourth, adverse-event reporting was not sufficiently uniform to support quantitative synthesis. In addition, the certainty of evidence across outcomes was not formally graded, which limits the strength of confidence that can be assigned to the pooled estimates. Finally, the review process itself was limited by English-language restriction, a small number of databases, and the absence of a formal certainty-of-evidence assessment. Review process limitations should also be acknowledged. The search was restricted to English-language publications and three major databases, and the number of eligible trials was small. In addition, the review was not prospectively registered, and the certainty of evidence was not formally graded across outcomes. These factors may limit transparency and the strength of confidence that can be placed in the pooled estimates.

### 4.3. Implications for Research

Future work should aim at direct head-to-head comparisons of anabolic agents in truly comparable, very high fracture risk populations and not indirect or sequential comparisons in heterogeneous cohorts. Studies are also needed to define the optimum duration of anabolic therapy and which, if any, subsequent antiresorptive agents, and for how long, most effectively maintain skeletal gains. Persisting evidence gaps exist for men and younger patients with glucocorticoid-induced osteoporosis—groups that are regularly excluded despite a high risk of fracture. Better patient selection with more-sophisticated skeletal evaluation tools, such as trabecular bone score and high-resolution peripheral quantitative computed tomography, may provide a further selection of those individuals who may be likely to benefit from the anabolic-first approach [32].

## 5. Conclusions

This systematic review and meta-analysis support an anabolic-first strategy in postmenopausal women at high or very high fracture risk. Initial treatment with teriparatide, abaloparatide, or romosozumab was associated with lower vertebral, clinical, non-vertebral, and hip fracture risk than comparator approaches, and the benefit of early anabolic therapy appeared to be maintained when followed by antiresorptive treatment. Despite heterogeneity among trials, the overall direction of effect was consistent across major fracture outcomes. In patients with recent fragility fractures, multiple vertebral fractures, or very low bone mineral density, anabolic-first sequencing should be strongly considered to reduce imminent fracture risk.

## Figures and Tables

**Table 1:** Characteristics and role of all included studies (*n* = 6).

Study (Author, Year) & Reference	Study Design	Population	Relevant Intervention/Comparator	Role in This Analysis	Primary Data for Meta-Analysis?
Neer et al., 2001 [14]	RCT, DB, PC	PM women with prevalent VF	Teriparatide vs. Placebo	PTH analog vs. placebo in very high-risk	Yes (vertebral, non-vertebral)
Miller et al., 2016 [15]	RCT, DB, PC (ACTIVE)	PM women with low BMD + fracture	Abaloparatide vs. Placebo	PTH analog vs. placebo in high/very high risk	Yes (vertebral, non-vertebral)
Bone et al., 2018 [16]	Extension RCT (ACTIVExtend)	ACTIVE completers	Abaloparatide → Alendronate vs. Placebo → Alendronate	Anabolic-first sequence vs. delayed anabolic	Yes (cumulative vertebral)
Kendler et al., 2018 [17]	RCT, DB, DD, AC (VERO)	PM women with severe OP (≥2 VF)	Teriparatide vs. Risedronate	Head-to-head vs. potent antiresorptive	Yes (vertebral, clinical, non-vertebral)
Saag et al., 2017 [18]	RCT, DB, AC (ARCH)	PM women with prevalent FX	Romosozumab → Alendronate vs. Alendronate → Alendronate	Anabolic-first sequence vs. antiresorptive-only	Yes (vertebral, clinical, hip, non-vertebral)
Cosman et al., 2016 [19]	RCT, DB, PC (FRAME)	PM women with high risk (T-score −2.5 to −3.5)	Romosozumab vs. Placebo (both → Denosumab)	Sclerostin inhibitor vs. placebo; context for sequence	Yes (vertebral)

Abbreviations: AC = active comparator, DB = double-blind, DD = double-dummy, FX = fracture, OP = osteoporosis, PC = placebo-controlled, PM = postmenopausal, RCT = randomized controlled trial, VF = vertebral fracture.

## Data Availability

The original contributions presented in this study are included in the article/Supplementary Material. Further inquiries can be directed to the corresponding author(s).

## Supplementary Materials

The following supporting information can be downloaded at https://www.mdpi.com/article/10.3390/medicina62040687/s1, PRISMA 2020 Checklist.

## Appendix A. Search Strategy

**Table A1:** Literature search strategy overview.

Component	Description
**Databases searched**	PubMed/MEDLINE, Embase, Cochrane Central Register of Controlled Trials (CENTRAL)
**Date of search**	From database inception to 31 January 2026
**Language restriction**	English
**Population**	Adults (≥18 years)
**Study design filter**	Randomized controlled trials (RCTs), controlled clinical trials
**Additional sources**	Manual screening of reference lists from included articles and relevant systematic reviews/guidelines (e.g., AACE, ESCEO)
**Synthesis approach**	Quantitative meta-analysis following PRISMA 2020 guidelines

**Table A2:** Search concepts and keywords (PubMed/MEDLINE).

Conceptual Domain	Keywords and Search Terms (MeSH + Free Text)
**Population: Osteoporosis**	“Osteoporosis”[Mesh] OR “Osteoporosis, Postmenopausal”[Mesh] OR osteoporos*[tiab] OR “bone loss”[tiab] OR “low bone mass”[tiab]
**Intervention: Anabolic agents**	“Bone Density Conservation Agents”[Mesh:Pharmacological Action] OR “Anabolic Agents”[Mesh] OR “Teriparatide”[Mesh] OR “abaloparatide”[Supplementary Concept] OR “Romosozumab”[Supplementary Concept] OR anabolic*[tiab] OR teriparatide[tiab] OR abaloparatide[tiab] OR romosozumab[tiab] OR PTH[tiab] OR “parathyroid hormone”[tiab] OR Tymlos[tiab] OR Evenity[tiab]
**Context: Very high fracture risk**	“Fractures, Bone”[Mesh] OR fracture*[tiab] OR “fragility fracture”[tiab] OR “vertebral fracture”[tiab] OR “hip fracture”[tiab] OR “imminent risk”[tiab] OR “high risk”[tiab] OR “severe osteoporosis”[tiab] OR “multiple fracture*”[tiab] OR “treatment failure”[tiab] OR “Bone Density”[Mesh] OR “bone mineral density”[tiab] OR BMD[tiab] OR T-score[tiab]
**Study design**	“Randomized Controlled Trial”[Publication Type] OR “Controlled Clinical Trial”[Publication Type] OR randomized[tiab] OR randomized[tiab] OR placebo[tiab] OR “clinical trial”[tiab] OR trial[tiab]

**Sample PubMed Search String (Adapted for Each Database):** #1 “Osteoporosis”[Mesh] OR osteoporos*[tiab] OR “bone loss”[tiab]. #2 “Anabolic Agents”[Mesh] OR “Teriparatide”[Mesh] OR anabolic*[tiab] OR teriparatide[tiab] OR abaloparatide[tiab] OR romosozumab[tiab]. #3 “Fractures, Bone”[Mesh] OR fracture*[tiab] OR “fragility fracture”[tiab] OR “vertebral fracture”[tiab]. #4 “high risk”[tiab] OR “severe osteoporosis”[tiab] OR “multiple fracture*”[tiab] OR “imminent risk”[tiab] OR “bone mineral density”[tiab] OR BMD[tiab]. #5 “Randomized Controlled Trial”[pt] OR randomized[tiab] OR randomized[tiab] OR placebo[tiab]. #6 #1 AND #2 AND #3 AND #4 AND #5. #7 Filters: English, Humans, Adult (19+ years).

## References

[1] Van Oostwaard M. (2018). Osteoporosis and the Nature of Fragility Fracture: An Overview. Perspectives in Nursing Management and Care for Older Adults.

[2] Salari N., Ghasemi H., Mohammadi L., Behzadi M.H., Rabieenia E., Shohaimi S., Mohammadi M. (2021). Global prevalence of osteoporosis according to the World Health Organization diagnostic criteria: A systematic review and meta-analysis. Arch. Osteoporos..

[3] World Health Organization (2021). Fragility Fractures: Global Burden and Epidemiology.

[4] Kanis J.A., Harvey N.C., McCloskey E., Bruyère O., Veronese N., Lorentzon M., Cooper C., Rizzoli R., Adib G., Al-Daghri N. (2020). Algorithm for the management of patients at low, high and very high risk of osteoporotic fractures. Osteoporos. Int..

[5] Qaseem A., Forciea M.A., McLean R.M., Denberg T.D. (2017). Treatment of low bone density or osteoporosis to prevent fractures in men and women. Ann. Intern. Med..

[6] McClung M.R. (2021). Role of bone-forming agents in the management of osteoporosis. Aging Clin. Exp. Res..

[7] Johansson H., Siggeirsdóttir K., Harvey N.C., Odén A., Gudnason V., McCloskey E., Sigurdsson G., Kanis J.A. (2017). Imminent risk of fracture after fracture. Osteoporos. Int..

[8] Camacho P.M., Petak S.M., Binkley N., Diab D.L., Eldeiry L.S., Farooki A., Harris S.T., Hurley D.L., Kelly J., Lewiecki E.M. (2020). American Association of Clinical Endocrinologists/American College of Endocrinology Clinical Practice Guidelines for the Diagnosis and Treatment of Postmenopausal Osteoporosis-2020 Update. Endocr. Pract..

[9] Kanis J.A., Cooper C., Rizzoli R., Reginster J.-Y., Scientific Advisory Board of the European Society for Clinical and Economic Aspects of Osteoporosis (ESCEO), Committees of Scientific Advisors and National Societies of the International Osteoporosis Foundation (IOF) (2019). European guidance for the diagnosis and management of osteoporosis in postmenopausal women. Osteoporos. Int..

[10] Cosman F., Nieves J.W., Dempster D.W. (2017). Anabolic therapies for osteoporosis. J. Bone Miner. Res..

[11] Lim S.Y., Bolster M.B. (2022). Clinical Utility of Romosozumab in the Management of Osteoporosis: Focus on Patient Selection and Perspectives. Int. J. Womens Health.

[12] Eastell R., Rosen C.J., Black D.M., Cheung A.M., Murad M.H., Shoback D. (2019). Pharmacological management of osteoporosis in postmenopausal women: An Endocrine Society clinical practice guideline. J. Clin. Endocrinol. Metab..

[13] Page M.J., McKenzie J.E., Bossuyt P.M., Boutron I., Hoffmann T.C., Mulrow C.D., Shamseer L., Tetzlaff J.M., Akl E.A., Brennan S.E. (2021). The PRISMA 2020 statement: An updated guideline for reporting systematic reviews. BMJ.

[14] Neer R.M., Arnaud C.D., Zanchetta J.R., Prince R., Gaich G.A., Reginster J.Y., Hodsman A.B., Eriksen E.F., Ish-Shalom S., Genant H.K. (2001). Effect of parathyroid hormone (1-34) on fractures and bone mineral density in postmenopausal women with osteoporosis. N. Engl. J. Med..

[15] Miller P.D., Hattersley G., Riis B.J., Williams G.C., Lau E., Russo L.A., Alexandersen P., Zerbini C.A.F., Hu M.-Y., Harris A.G. (2016). Effect of abaloparatide vs. placebo on new vertebral and nonvertebral fractures in postmenopausal women with osteoporosis: A randomized clinical trial. JAMA.

[16] Bone H.G., Cosman F., Miller P.D., Williams G.C., Hattersley G., Hu M.Y., A Fitzpatrick L., Mitlak B., Papapoulos S., Rizzoli R. (2018). ACTIVExtend: 24 months of alendronate after 18 months of abaloparatide or placebo for postmenopausal osteoporosis. J. Clin. Endocrinol. Metab..

[17] Kendler D.L., Marin F., Zerbini C.A.F., Russo L.A., Greenspan S.L., Zikan V., Bagur A., Malouf-Sierra J., Lakatos P., Fahrleitner-Pammer A. (2018). Effects of teriparatide and risedronate on new fractures in postmenopausal women with severe osteoporosis (VERO): A multicentre, double-blind, double-dummy, randomised controlled trial. Lancet.

[18] Saag K.G., Petersen J., Brandi M.L., Karaplis A.C., Lorentzon M., Thomas T., Maddox J., Fan M., Meisner P.D., Grauer A. (2017). Romosozumab or alendronate for fracture prevention in women with osteoporosis. N. Engl. J. Med..

[19] Cosman F., Crittenden D.B., Adachi J.D., Binkley N., Czerwinski E., Ferrari S., Hofbauer L.C., Lau E., Lewiecki E.M., Miyauchi A. (2016). Romosozumab treatment in postmenopausal women with osteoporosis. N. Engl. J. Med..

[20] Tian A., Jia H., Zhu S., Lu B., Li Y., Ma J., Ma X. (2021). Romosozumab versus Teriparatide for the Treatment of Postmenopausal Osteoporosis: A Systematic Review and Meta-analysis through a Grade Analysis of Evidence. Orthop. Surg..

[21] Compston J.E., McClung M.R., Leslie W.D. (2019). Osteoporosis. Lancet.

[22] Bandeira L., Lewiecki E.M. (2022). Anabolic therapy for osteoporosis: Update on efficacy and safety. Arch. Endocrinol. Metab..

[23] Kanis J.A., Johansson H., McCloskey E., Liu E., Åkesson K., Anderson F., Azagra R., Bager C., Beaudart C., Bischoff-Ferrari H. (2023). Previous fracture and subsequent fracture risk: A meta-analysis to update FRAX. Osteoporos. Int..

[24] Leder B.Z., Tsai J.N., Uihlein A.V., Burnett-Bowie S.A., Zhu Y., Foley K., Lee H., Neer R.M. (2014). Two years of Denosumab and teriparatide administration in postmenopausal women with osteoporosis (The DATA Extension Study): A randomized controlled trial. J. Clin. Endocrinol. Metab..

[25] Tsai J.N., Uihlein A.V., Lee H., Kumbhani R., Siwila-Sackman E., McKay E.A., Burnett-Bowie S.A., Neer R.M., Leder B.Z. (2013). Teriparatide and denosumab, alone or combined, in women with postmenopausal osteoporosis: The DATA study randomised trial. Lancet.

[26] Cosman F., Langdahl B., Leder B.Z. (2024). Treatment sequence for osteoporosis. Endocr. Pract..

[27] Langdahl B.L., Teglbjærg C.S., Ho P., Chapurlat R., Czerwinski E., Kendler D.L., Reginster J., Kivitz A., Lewiecki E.M., Miller P.D. (2015). A 24-Month study evaluating the efficacy and safety of denosumab for the treatment of men with low bone mineral density: Results from the ADAMO trial. J. Clin. Endocrinol. Metab..

[28] Hiligsmann M., Reginster J.Y., Tosteson A.N.A., Bukata S.V., Saag K.G., Gold D.T., Halbout P., Jiwa F., Lewiecki E.M., Pinto D. (2019). Recommendations for the conduct of economic evaluations in osteoporosis: Outcomes of an experts’ consensus meeting organized by the European Society for Clinical and Economic Aspects of Osteoporosis, Osteoarthritis and Musculoskeletal Diseases (ESCEO) and the US branch of the International Osteoporosis Foundation. Osteoporos. Int..

[29] Liu L., Wu S., Wei L., Xia Z., Ji J., Huang D. (2025). Romosozumab adverse event profile: A pharmacovigilance analysis based on the FDA Adverse Event Reporting System (FAERS) from 2019 to 2023. Aging Clin. Exp. Res..

[30] Orwoll E.S. (1998). Osteoporosis in men. Endocrinol. Metab. Clin. N. Am..

[31] Langdahl B.L., Silverman S., Fujiwara S., Saag K., Napoli N., Soen S., Enomoto H., Melby T.E., Disch D.P., Marin F. (2018). Real-world effectiveness of teriparatide on fracture reduction in patients with osteoporosis and comorbidities or risk factors for fractures: Integrated analysis of 4 prospective observational studies. Bone.

[32] Hans D., Šteňová E., Lamy O. (2017). The Trabecular Bone Score (TBS) Complements DXA and the FRAX as a Fracture Risk Assessment Tool in Routine Clinical Practice. Curr. Osteoporos. Rep..

